# Recent advances in Ti_3_C_2_T_*x*_ MXene-based composites for electrocatalytic applications

**DOI:** 10.1039/d5na01003a

**Published:** 2026-02-06

**Authors:** Minh-Hai Tran, Vo Thi Thuy Linh, Ly Tan Nhiem, Phan Khanh Thinh Nguyen, Qui Thanh Hoai Ta

**Affiliations:** a Department of Materials and Chemical Engineering, Concordia University 1515 Ste. Catherine St. W. Montreal QC H3G 2W1 Canada mis.miha@gmail.com; b Faculty of Natural Science Education, Pham Van Dong University Quang Ngai Vietnam vttlinh@pdu.edu.vn; c Faculty of Chemical and Food Technology, Ho Chi Minh City University of Technology and Education 01 Vo Van Ngan Street, Thu Duc Ward Ho Chi Minh City Vietnam nhiemlt@hcmute.edu.vn; d School of Chemical, Biological, and Battery Engineering, Gachon University 1342 Seongnamdaero, Sujeong-gu Seongnam-si Gyeonggi-do 13120 Republic of Korea thinhnpk@gachon.ac.kr; e Institute of Advanced Technology, Vietnam Academy of Science and Technology 1B TL29 Street, An Phu Dong Ward Ho Chi Minh City 700000 Vietnam tathanhhoaiqui2292@gmail.com

## Abstract

Electrocatalysis is central to the development of sustainable energy conversion technologies and environmental remediation systems, yet the rational design of high-performance, cost-effective, and durable electrocatalysts remains a fundamental challenge. In recent years, MXenes, a rapidly expanding family of two-dimensional transition-metal carbides, nitrides, and carbonitrides, have emerged as a transformative class of materials owing to their metallic conductivity, tunable surface chemistry, rich termination groups, and structural versatility. These unique attributes endow MXenes with exceptional potential for a wide spectrum of electrocatalytic reactions, including the hydrogen evolution reaction, oxygen evolution reaction, and CO_2_ reduction reaction. This review provides a fundamental assessment of Ti_3_C_2_T_*x*_ MXene-based electrocatalysts, encompassing synthesis strategies, surface and termination chemistry, and structure–performance relationships. Particular emphasis is placed on how etching routes, delamination processes, and functional group engineering govern catalytic performances. Recent advances in Ti_3_C_2_T_*x*_ MXene composites, heterostructures, and defect engineering are systematically analyzed to elucidate synergistic effects and catalytic enhancement mechanisms. Furthermore, the challenges hindering practical implementation, such as oxidation instability, restacking, ion transport limitations, and the need for scalable manufacturing, are discussed. By integrating experimental insights with theoretical modeling and emerging data-driven approaches, this review outlines future research directions and design principles aimed at bridging the gap between laboratory-scale performance and industrial deployment. Overall, this work is expected to establish Ti_3_C_2_T_*x*_ MXene-based composites as a versatile and continuously evolving platform for next-generation electrocatalysis, while providing a strategic roadmap for their rational design and development in electrocatalytic applications.

## Abstract 2

Electrocatalysis underpins sustainable energy conversion and environmental remediation, yet designing high-performance, affordable, and durable electrocatalysts remains a critical challenge.

## Graphical abstract

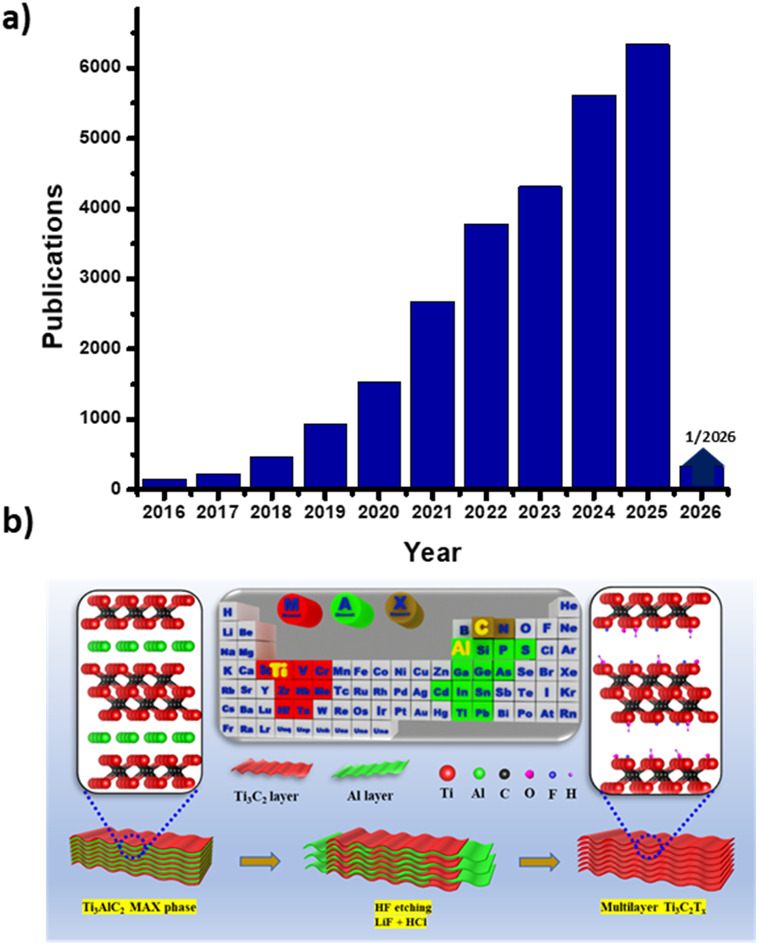

## 1. Introduction

In contemporary society, increasing concerns over the energy crisis and global warming have emerged, largely driven by rapid population growth and industrialization. To address these pressing challenges, it is imperative to explore alternative energy sources to replace fossil fuels and to develop strategies that mitigate environmental degradation.^1–3^ The scale-up synthesis of green energy carriers, including ammonia, solar, and hydrogen energy, as alternatives to fossil fuels, is strongly encouraged. In particular, green energy conversion strategies such as electrochemical water splitting and carbon dioxide capture and conversion are central to achieving carbon neutrality. Among the potential technologies, electrocatalysis has emerged as a rising star in the field of energy storage and conversion, playing a crucial role in promoting sustainable development. However, conventional electrocatalysts based on noble metals (Ir, Au, and Pt) suffer from high cost, limited availability, and poor stability and durability under harsh operating conditions, which severely restrict their large-scale application at the pilot level.^4,5^ Therefore, the development of low-cost electrocatalysts with high efficiency is imperative to advance the new era of green development and sustainability.^6–8^ Material-based catalysis has garnered significant interest within the scientific community for its potential in energy harvesting, particularly in the development of high-performance electrocatalysts. Materials such as graphene, MXenes, conductive polymers, and carbon-based structures have been extensively investigated for their applications in metal-ion batteries (*e.g.*, sodium- and lithium-ion batteries), the hydrogen evolution reaction (HER), the oxygen reduction reaction (ORR), and supercapacitors.^9–13^

MAX phases, the precursors to MXenes, possess the general formula M_*n*+1_AX_*n*_, where *n* ranges from 1 to 4. In this structure, M represents early transition metals such as chromium (Cr), titanium (Ti), hafnium (Hf), molybdenum (Mo), niobium (Nb), or vanadium(V); A is typically a group 13 or 16 element, such as aluminum (Al) or silicon (Si); and X denotes carbon and/or nitrogen.^14^ The A element usually has a lower melting point than both M and X and is bonded to the M layers *via* metallic interactions.^15,16^ In contrast, the bonding between M and X involves a combination of covalent, metallic, and ionic interactions, which are generally stronger than those between M and A. MXenes are synthesized by selectively etching the A layer from MAX phases, resulting in two-dimensional transition metal carbides, nitrides, or carbonitrides.^17,18^ After the etching process, MXene surfaces are decorated with terminal functional groups (T_*x*_), which play a crucial role in tailoring interfacial interactions and enabling diverse composite fabrication and applications. MXene-based composites have been widely applied in diverse fields, including biomedical applications and environmental remediation. For instance, Cu-doped MXene has been confirmed to exhibit strong antibacterial activity against *Escherichia coli* and *Staphylococcus aureus*, achieving inhibition and bacterial killing efficiencies of approximately 99%.^19^ The optimized Cu-MXene generates reactive oxygen species (ROS), which damage bacterial DNA and disrupt cellular membranes. Moreover, Cu-MXene interferes with the bacterial electron transport chain, thereby inhibiting essential metabolic activities. The nanoscale size and positively charged surface of Cu-MXene play a crucial role in enhancing adhesion to bacterial cell surfaces, further improving its antibacterial effectiveness. In another study, Cu/MXene was designed for urea adsorption, exhibiting an adsorption capacity in the range of 39.3–78.6 µmol.^20^ The plausible adsorption mechanism was attributed to the uniform dispersion of Cu on the MXene surface and the strong interaction between urea molecules and Cu sites, where the electron-rich oxygen atom of the carbonyl group in urea coordinates with electron-deficient Cu species. This structural feature highlights the promising potential of MXene-based materials as dialysis membranes for wastewater treatment. Cetyltrimethylammonium bromide (CTAB) and polyvinylpyrrolidone (PVP) have been incorporated with MXenes to achieve adjustable permeability in membrane-based systems.^21^ The optimized composites exhibited high adsorption capacities toward organic dyes, with loading amounts of methyl orange and methylene blue reaching 108.61 and 70.05 mg g^−1^, respectively. In the same context, PVP/MXene composites have been synthesized for energy-harvesting applications. The resulting PVP/MXene electrode exhibited a high specific capacitance of 154.6 F g^−1^ at a current density of 0.3 A g^−1^.^22^ The optimized interlayer spacing of the MXene, together with the uniform wrapping of PVP, plays a key role in increasing the number of accessible electroactive sites and enhancing electron transport within the electrode material in aqueous electrolytes.

**Fig. 1:**
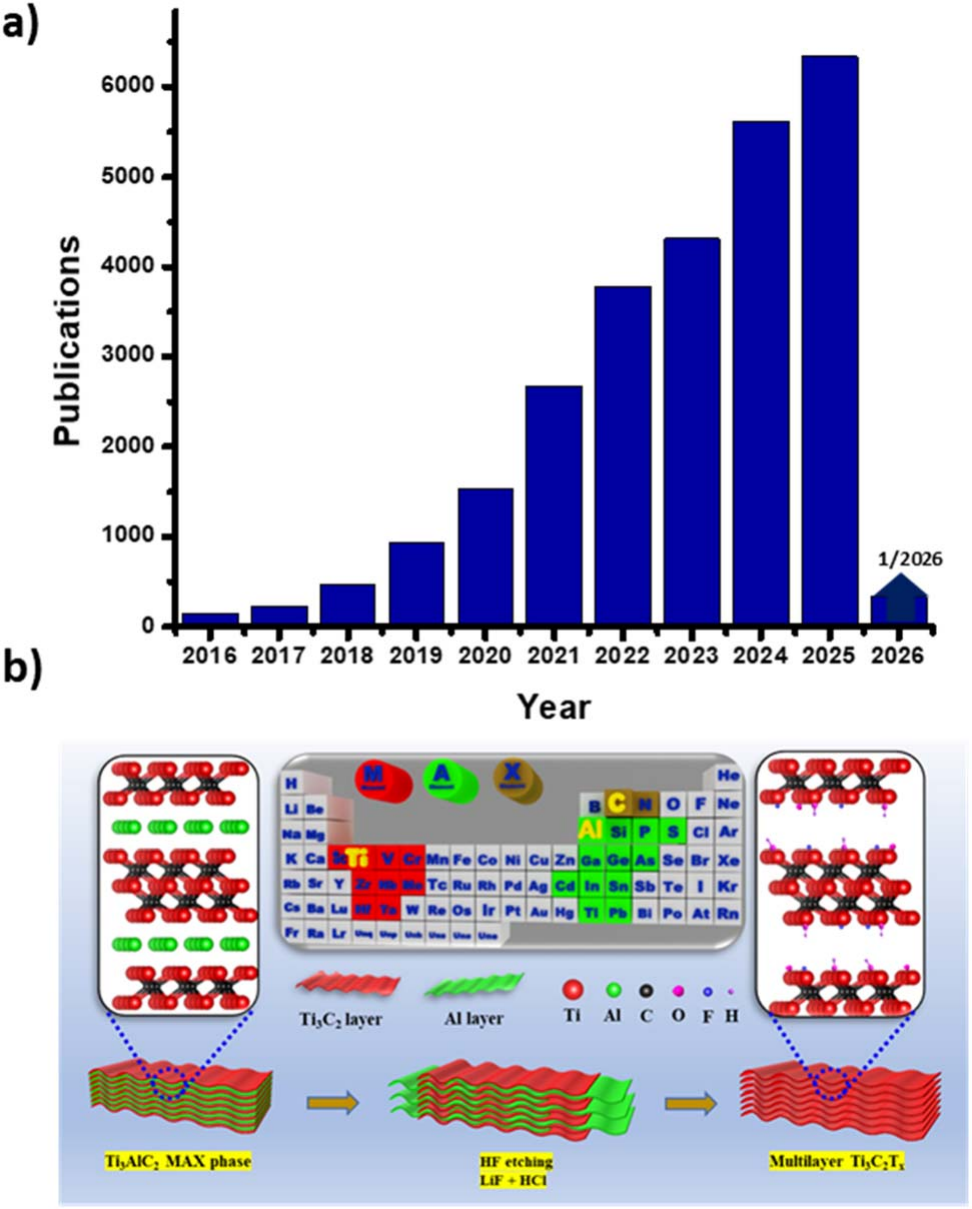
Animated illustration showing (a) the evolution of MXene-related publications and (b) the fluorine-based synthesis routes for Ti_3_C_2_T_*x*_ MXene. Reproduced from ref. 31 with permission from Elsevier, copyright 2021.

As shown in Fig. 1a, Web of Science records show a rapidly increasing number of publications focusing on MXenes and their applications, with annual outputs growing steadily. Although numerous studies have reported the electrochemical performance of MXenes in various applications,^23–26^ a review that systematically summarizes and discusses the fundamental principles and recent progress in the HER, OER, and CO_2_RR over MXenes and their derivatives is still urgently needed.

Since its discovery in 2011, Ti_3_C_2_T_*x*_ MXene has attracted considerable attention due to its unique physicochemical properties, including a multilayered structure with abundant surface-active sites, excellent electrical conductivity, and intrinsic hydrophilicity. This review aims to elucidate the fundamental aspects of MXenes, including their synthesis methods, physicochemical properties, and potential applications. Recent advancements in the electrocatalytic applications of MXenes are highlighted, followed by a critical analysis of current findings and a forward-looking perspective on the future development of MXene-based catalysts.

## 2. Synthesis methods

Ti_3_C_2_T_*x*_ MXenes are two-dimensional (2D) transition metal carbides and nitrides that show unique electrical conductivity, excellent thermal conductivity, a large surface area with abundant surface termination groups, and superior mechanical strength. The first preparation of MXenes was reported in 2011 using hydrofluoric acid (HF) as the etchant for the MAX phase. Due to their unique physicochemical properties, MXenes have emerged as promising materials for a wide range of applications, including sensors, lithium-ion batteries, biomedicine, solar cells, electromagnetic interference shielding, gas sensors, and supercapacitors. Since then, extensive investigations have been conducted on MXenes across various fields. Notably, significant progress has been achieved in utilizing MXenes for electrocatalytic technologies.^27–29^

The HF etching technique offers facile access to MXene synthesis at mild reaction temperatures (Fig. 1b). It involves the selective removal of the Al layer from the MAX phase using HF, followed by shaking or sonication, resulting in a nanosheet structure.^30^

**Fig. 2:**
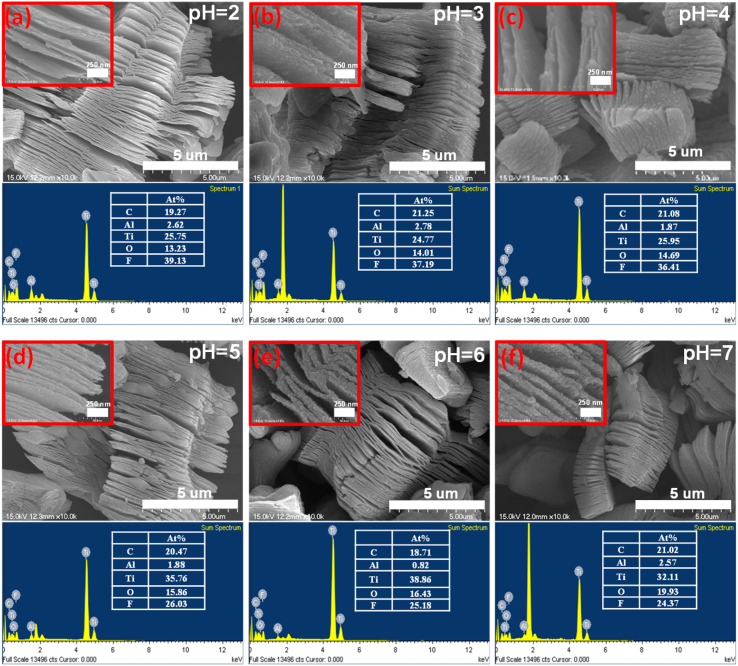
(a–f) SEM images of Ti_3_C_2_T_*x*_ MXene synthesized *via* HF etching, illustrating the morphology at different pH values after the washing process. Reproduced from ref. 18 with permission from Elsevier, copyright 2021.

The resulting accordion-like morphology necessitates subsequent delamination to obtain monolayer or few-layer MXenes, which typically requires a more sophisticated process. Given the hazardous nature and high toxicity of HF, a safer alternative has been developed through *in situ* HF etching by combining hydrochloric acid (HCl) with lithium fluoride (LiF).^32^ In this approach, HCl reacts with LiF to generate *in situ* HF, while the resulting Li^+^ ions intercalate between the MXene layers, increasing interlayer spacing and facilitating the formation of monolayer or few-layered MXenes.^33^ The presence of F^−^ ions in the etching solution poses significant environmental risks. In terms of surface functionalization, MXenes with a high concentration of fluorine groups are detrimental to their energy storage activities. It has been demonstrated that fluorine-free MXenes exhibit superior structural stability and lower environmental hazards compared to conventionally etched MXenes.^34,35^ Among the emerging etching strategies, the molten salt method has shown promise in producing desired surface F-free termination groups and scaling up the preparation of MXenes. The synergistic effect of multilayered morphology and the bonding between Ti–C layers significantly influences the electrical properties of MXenes (Fig. 2). Interface scattering can be minimized due to electron transmission between layers, facilitated by the presence of metal d-electrons. Additionally, the surface termination groups (T_*x*_) can modulate electron density and contribute to pseudocapacitive behavior through faradaic reactions, thereby enhancing both electrical conductivity and energy storage capabilities.^36,37^

The properties of MXenes are influenced by both surface termination groups and unsaturated metal sites, which provide active sites and electrostatic charge on the MXene surface. MXenes exhibit excellent mechanical properties, including high strength and flexibility, attributed to the presence of covalent bonds and *van der Waals* interactions.^38^ These characteristics enable their application in both rigid and flexible systems, such as wearable electronics and structural reinforcement. Moreover, their broad-spectrum light absorption facilitates applications in photocatalytic activities through surface plasmon resonance effects.

The electrical properties of MXenes are critical to their functional applications and are significantly influenced by the material type and synthesis methods, which determine their morphology, surface termination groups, and lateral dimensions. Maleski and co-workers reported that the lateral size of MXene nanosheets impacts both optical and electrical properties.^39^ Specifically, films fabricated from large-sized MXenes exhibited a peak conductivity of approximately 5.000 S cm^−1^, compared to around 1.000 S cm^−1^ for those derived from smaller MXene nanosheets. This enhancement is attributed to the more compact stacking of larger flakes, which reduces interlayer gaps and improves charge transport.

Furthermore, MXenes synthesized using traditional HF-based etching methods show higher electrical conductivity than that produced *via in situ* HF generation. In terms of surface chemistry, MXenes with oxygen (O) terminations demonstrate lower electrical conductivity than their hydroxyl (OH)-terminated and unmodified counterparts. Additionally, MXenes treated under an argon atmosphere exhibit reduced conductivity relative to those treated with ammonia. This difference is likely due to the nitridation process in ammonia, which helps preserve the electron-rich structure, thereby enhancing conductivity.^40,41^

## 3. Properties of Ti_3_C_2_T_*x*_

MXenes exhibit excellent mechanical properties owing to the robust covalent bonding between the M and C layers. Upon removal of the Al layer, the strength of the M–C bonds is further enhanced due to the high elastic constant, particularly when functionalized with surface termination groups.^42,43^ It has been confirmed that nitride-based MXenes possess a higher Young's modulus than carbide-based MXenes, which can be attributed to the higher electronegativity of nitrogen compared to carbon. Surface terminations also significantly influence the strength of M–X bonding. Among the functional groups, O-terminated MXenes demonstrate higher mechanical strength but lower electrical conductivity than OH-terminated counterparts.^44–46^ This behavior can be explained by the strong covalent bonds formed between oxygen and the M layer, which reduce the number of free electrons available to facilitate electrical conduction.

The electronic properties of MXenes are strongly influenced by the number of layers, the nature of the energy band gap (direct or indirect), and the type of surface termination groups. Monolayer MXenes typically exhibit larger band gap values compared to their multilayer counterparts, and it is evident that monolayers generally possess an indirect band gap. Surface terminations T_*x*_ play a crucial role in determining the metallic or semiconducting behavior of MXenes. While pristine MXene nanostructures exhibit metallic characteristics, they can be transformed into semiconductors through appropriate functionalization.

Density functional theory (DFT) calculations reveal that –OH-terminated and –F-terminated MXenes exhibit band gaps of approximately 0.1 eV and 0.05 eV, respectively.^47^ These narrow band gaps ensure high electron mobility, thereby enhancing electrocatalytic performance. Furthermore, substituting Ti with Mo to form Mo_2_TiC_2_(OH)_2_ has been reported to yield an energy band gap as low as ∼0.05 eV.^48^

**Fig. 3:**
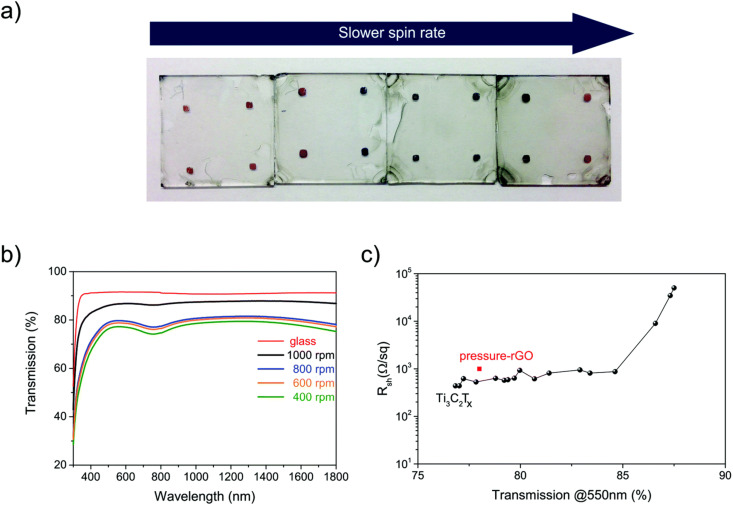
(a) Ti_3_C_2_T_*x*_ MXene films were deposited on glass slides along with (b and c) their corresponding transmission spectra and sheet resistance. Reproduced from ref. 49.

MXenes exhibit a wide range of optical behaviors, including absorption, refractive index, and reflectivity. Specifically, Ti_3_C_2_T_*x*_ MXene demonstrates approximately 92% transmittance at wavelengths below 500 nm and about 77% at 550 nm.^42,49^ As shown in Fig. 3a and b, the samples exhibit a transmission of approximately 86% in both the visible (VIS) and near-infrared (NIR) regions. The intrinsic absorption of the glass substrate is evident from the dip in transmission below 390 nm. Furthermore, the sheet resistance increases from 400 to 700 Ω sq^−1^ with enhanced transparency, which can be attributed to reduced layer overlap and weaker flake networking at the grain boundaries.^49^ This phenomenon suggests that MXene holds great potential for photoelectrochemical and optoelectronic applications. The optical properties of MXene can be effectively tuned by adjusting its thickness, surface functional groups, and intercalated ions. For instance, treatment with tetramethylammonium hydroxide enhances the transmittance from 75% to 92%, whereas urea and hydrazine reduce the transmittance.^46^

One of the major disadvantages of Ti_3_C_2_T_*x*_ MXene is its susceptibility to oxidation under high-humidity ambient conditions. MXene can be oxidized to form TiO_2_ in oxygen- and moisture-rich environments.^50^ Larger-sized MXene flakes are more resistant to oxidation compared to smaller ones. Furthermore, elevated temperature, high concentrations of storage solvents, and UV illumination can accelerate the oxidation process.^51^ It has been demonstrated that MXene can be well dispersed in organic solvents, among which anhydrous isopropanol is more effective in preventing oxidation than water.^52,53^

To mitigate the oxidation of MXene under ambient conditions, antioxidation strategies such as the use of sodium l-ascorbate or polyanions have been employed.^54,55^ These treatments effectively preserve the physicochemical properties and the characteristic accordion-like morphology of MXene, allowing it to remain stable for up to three weeks when stored in a sodium l-ascorbate solution.

Based on DFT calculations, pristine MXene exhibits intrinsic magnetic properties, which can be significantly altered upon the introduction of functional groups. The magnetic behavior depends strongly on the type of transition metals and the nature of surface terminations. For instance, Ti_3_C_2_ and Ti_4_C_3_ are inherently magnetic MXenes, but they become non-magnetic once functional groups are introduced. This phenomenon can be explained by the formation of p–d bonding between the transition metal atoms and T_*x*_ groups, as well as orbital splitting effects.^56,57^ Specifically, the formation of an octahedral coordination cage around the metal layer leads to splitting of the d orbitals into the t_2_g (d_*xy*_, d_*yz*_, and d_*xz*_) and eg (d_*x*^2^−y^2^_ and d_*z*^2^_) manifolds. Since the t_2_g orbitals have lower energy than the eg orbitals, the electronic configuration of the d orbitals, and thus the magnetic properties, are modified.^56,58^

## 4. Potential application of Ti_3_C_2_T_*x*_-based electrocatalysts

**Fig. 4:**
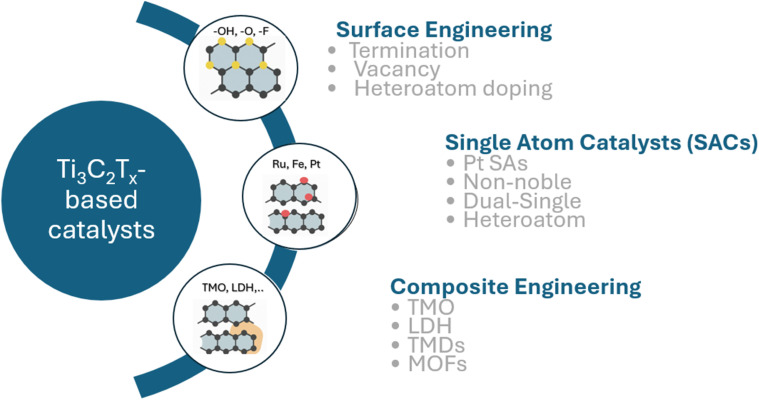
Overview of various material engineering strategies to improve the electrocatalytic performance of MXene-based systems.

Ti_3_C_2_T_*x*_ is a typical MXene, accounting for about 70% of related publications in the past decade due to its mechanical flexibility for practical applications and well-established, scalable synthesis from MAX phases. As a 2D layered structure, Ti_3_C_2_T_*x*_ possesses a large effective surface area with tunable interlayer spacing that adjusts binding to intermediates. It exhibits outstanding metallic conductivity (up to 10 000 S cm^−1^), enabling fast electron transfer. The terminal T_*x*_ groups render the surface hydrophilic, ensuring good electrolyte wetting.^59–61^ Specifically, during the etching stage, the surface becomes terminated with functional groups such as –O, –OH, and –F, which impart hydrophilicity and increase interlayer spacing, thereby enhancing the accessible area for reactions.^14,62^ The termination also plays an important role in boosting the electrocatalytic efficiency of Ti_3_C_2_T_*x*_ based on the tunability of electronic states and active sites, such as direct participation in the reaction or an anchor support for catalyst nanomaterials on the MXene surface.^63–65^ However, pristine Ti_3_C_2_T_*x*_ itself exhibits limited intrinsic activity and suboptimal binding energy.^66,67^ Recently, there has been a great effort to enhance the performance of Ti_3_C_2_T_*x*_-based electrocatalysts by tuning terminal groups, defect engineering, doping, interlayer engineering or creating heterostructures to anchor the active components and regulate the electronic structure, adjust binding energies and enhance stability, as illustrated in Fig. 4. Depending on the specific applications, their mechanisms and state of the art will be discussed further.

### 4.1 Hydrogen evolution reaction

The HER is a fundamental electrochemical process regarding the reduction at the cathode, where protons or water molecules are converted to hydrogen gas (H_2_). Hydrogen is known as an eco-friendly fuel due to its only water vapor exhaust, high efficiency and carbon-zero process. Water splitting is a promising method for hydrogen production that aligns with sustainability goals, with the HER serving as a key half-reaction in which protons and hydroxyl ions from water are converted to hydrogen gas. Conventionally, platinum (Pt) is well-known as a near-ideal catalyst for the HER, but Pt is expensive and scarce, posing challenges for wide adaptability. Therefore, there is a critical need to identify cost-effective alternatives to Pt without compromising catalytic efficiency. Superior to other 2D materials, MXenes have gained significant attention due to their excellent electrical conductivity and high surface area from nanolayers, which benefit electrocatalytic performance.^68^ However, their performance is generally lower than Pt-based systems due to the limited intrinsic activity and poor adsorption of hydrogen on the surface.^63,68,69^

**Fig. 5:**
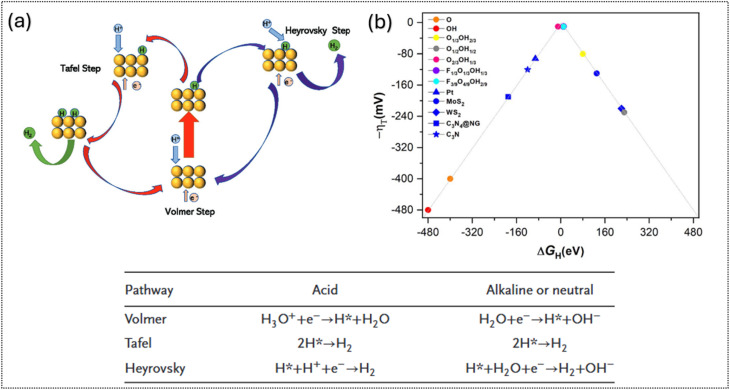
(a) Schematic of the hydrogen evolution mechanism on the electrocatalyst surface: blue = protons, green = hydrogen intermediates/molecules, yellow = metal atoms; the inset shows HER pathways in different electrolytes with the corresponding reaction steps. Reproduced under the terms of the CC-BY license.^71^ Copyright 2023, Wiley-VCH. (b) Volcano plot of theoretical overpotential (*η*) *vs.* Δ*G*_H_ for differently terminated Ti_3_C_2_T_*x*_ surfaces. Reproduced under the terms of the CC BY-NC license.^72^ Copyright 2023, RSC.

As shown in Fig. 5a, the hydrogen evolution reaction (HER) proceeds *via* either the Volmer–Tafel or Volmer–Heyrovsky pathway. Starting with the Volmer step, a proton (H^+^) in acidic media or a water molecule (H_2_O) in an alkaline environment is electrochemically reduced to an adsorbed hydrogen intermediate (H*) on the catalyst surface. Hydrogen gas (H_2_) is then formed either by the combination of two H* species in the Tafel step or by the reaction of H* with a proton and an electron in the Heyrovsky step. The Tafel slope can be used to identify the rate-determining step (RDS), the slowest elementary step that controls the overall reaction rate in the HER.^70^ Specifically,typical Tafel slopes of about 120, 40 and 30 mV dec^−1^ correspond to the limited kinetics of Volmer, Heyrovsky, and Tafel steps, respectively. These values provide insight into whether hydrogen adsorption, electrochemical desorption or chemical recombination controls the overall reaction rate. From the thermodynamic perspective, the hydrogen adsorption free energy (Δ*G*_H*_) is considered as a key parameter for HER activity. If the values of Δ*G*_H*_ are too positive, it will hinder proton adsorption, resulting in sluggish Volmer kinetics. Meanwhile if those values are too negative, it will impede hydrogen desorption, reflecting overly strong H* binding in the Tafel or Heyrovsky step. An ideal Δ*G*_H*_ is near 0 eV, ensuring a balance between adsorption and desorption for optimal HER performance.^71^ This ideal condition is commonly represented by the volcano-type relationship in Fig. 5b, where optimal catalysts lie near the peak and exhibit minimal overpotential. In the same figure, an overview of theoretical studies on MXenes with different surface terminations and other HER catalysts is presented. As indicated by the color-coded regions, functionalized MXenes occupy a more optimal position on the volcano plot relative to conventional HER catalysts. The blue region represents a comparison of different surface terminations, such as –O, –OH, and –F/–OH, demonstrating that surface functionalization is key to enhancing HER activity.

To date, several strategies have been proposed to achieve an optimal Δ*G*_H*_ for Ti_3_C_2_T_*x*_-based catalysts. These include surface engineering through tailoring the terminal functional groups or defect introduction to modulate the electronic structure. Increasing the density of active sites enhances the number of sites with near-optimal Δ*G*_H*_, thereby improving reaction kinetics. In addition, forming composites with transition metals or hydroxides promotes water dissociation during the Volmer step under alkaline conditions. The synergistic effect of composites not only increases the catalyst sites but also improves the catalyst stability during long-term operation. When combined, these approaches offer a comprehensive route to maximize HER performance in Ti_3_C_2_T_*x*_-based catalysts.^64,73–76^

One of the early studies related to surface engineering concerns the termination group (T_*x*_). Theoretical calculations of the change in the free energy for H adsorption (Δ*G*_H_) can be used to estimate the HER activity of a catalyst, with an ideal HER catalyst (Δ*G*_H_) value close to zero, where hydrogen adsorption and desorption are balanced, leading to fast Volmer and Tafel/Heyrovsky steps. Specifically, F^−^ ions are introduced into the titanium carbide MXene during MAX etching; however, fluorine has been shown to inhibit the HER activity of titanium carbide MXene due to unfavorable thermodynamics.^77^ In the theoretical study by the Illas team,^72^ DFT results to calculate Gibb's energy, combined with Pourbaix and Bader analysis, revealed that the terminations on Ti_3_C_2_ MXene differently influence hydrogen adsorption and desorption, thereby affecting the HER. For instance, F-termination binds H too weakly, leading to Volmer limitation. They recommended that tuning the ratio of O, OH, and F terminations is key to optimizing catalytic performance.

According to Handoko *at el*.,^78^ increasing F surface coverage on Ti_3_C_2_T_*x*_ leads to a marked decrease in HER activity. Meanwhile, fully O-terminated Ti_3_C_2_T_*x*_ exhibits the most favorable basal-plane coverage at zero applied potential, with a near-zero Δ*G*_H_ value comparable to that of Pt.^77^ In 2019, Jiang *et al.*^77^ proposed a method to fully functionalize Ti_3_C_2_T_*x*_ with oxygen (Ti_3_C_2_O_*x*_), resulting in enhanced HER performance, with an overpotential (*η*_10_) and Tafel slope of 190 mV and 60.7 mV dec^−1^, respectively, superior to the 266 mV and 109.8 mV dec^−1^ observed for Ti_3_C_2_T_*x*_. It demonstrates that the strategy of surface modification is an effective way for enhancing the catalytic performance of Ti_3_C_2_T_*x*_.

Single-atom catalysts (SACs) consist of mononuclear metal atoms anchored on support layers to maximize atomic efficiency, enabling nearly 100% atomic utilization while minimizing noble or transition metal loading. Beyond cost reduction, the defining advantage of SACs in the HER lies in their well-defined coordination environment and tunable electronic structure, which allow precise regulation of hydrogen adsorption and reaction kinetics at the molecular scale. Currently, in MXene-based SAC systems, various defect engineering strategies, such as metal or carbon vacancies and surface termination vacancies, heteroatom-doped coordination motifs, and strong metal–support interactions (SMSIs), are employed for these composites.

The enhancement of MXene catalysts by SACs can be attributed to synergistic mechanisms. Specifically, defect sites provide localized charge redistribution and unsaturated coordination environments, which generate strong binding energies between the metal atoms and the Ti_3_C_2_T_*x*_ substrate, stabilizing the atoms during electrochemical operation. This improves structural stability and helps maintain catalytic activity under prolonged HER conditions. The isolated metal atoms in SACs function as discrete active centers whose electronic states differ fundamentally from those of nanoparticles or bulk materials. Herein SACs offer a strong electronic coupling between the single atom and the MXene support, inducing charge transfer and d-band modulation of the metal center. That combination helps to adjust the hydrogen adsorption free energy (Δ*G*_H*_) toward the optimal value of approximately 0 eV in the volcano plot. Therefore, SACs can simultaneously facilitate hydrogen adsorption in the Volmer step and hydrogen desorption in the subsequent Heyrovsky or Tafel step, which overcomes kinetic limitations commonly observed in conventional catalysts.

Furthermore, in alkaline HER, where water dissociation is often the rate-determining step, SAC-supported MXenes can exhibit bifunctional reaction sites. The single metal atom preferentially adsorbs hydrogen intermediates (H*), while adjacent Ti–O or Ti–OH species on the MXene surface promote H_2_O activation and OH^−^ desorption. An innovation of N-coordination anchoring also has been proved in SAC synthesis to improve the stability of metal adatoms on C–M–N structure motifs. It is explained by the ability to create multi-point anchoring (M–N and M–C interactions), raise the migration energy barrier for the metal atom and suppress sintering and leaching problems of SACs. As the stability of SACs is affected by the dynamic coordination environment, it is noted that this composite catalyst is only suitable for the HER and not for anodic reactions. In addition, during HER operation, surface terminations can be rearranged, and the Ti surface can undergo partial reconstruction, which may alter metal–support interactions and change the coordination geometry of the SAC over time.

**Fig. 6:**
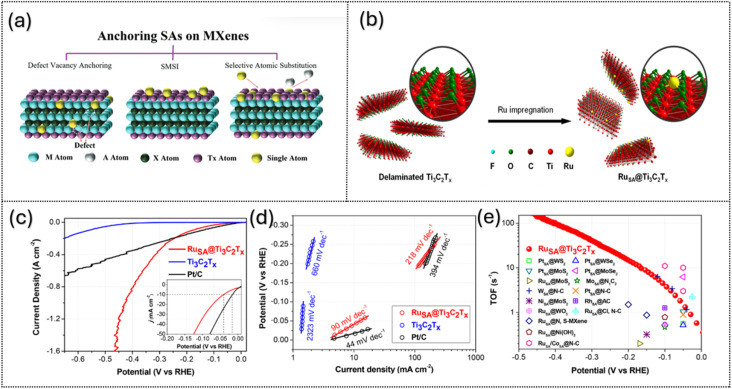
(a) Illustration of three pathways for anchoring single metal atoms on Ti_3_C_2_T_*x*_-MXene. Reproduced under the terms of the CC-BY license.^63^ Copyright 2025, Wiley-VCH. (b–e) Schematic illustration of RuSA@Ti_3_C_2_T_*x*_ synthesis and its hydrogen evolution reaction (HER) performance. Reproduced under the terms of the CC-BY license. Copyright 2023, Wiley.

Various synthetic strategies, including wet-chemical impregnation, atomic trapping, and vapor-phase or electrochemical deposition, have been employed to construct SACs with controlled coordination environments, as illustrated in Fig. 6a. These methods prevent aggregation and stabilize the single atoms through strong interactions such as covalent bonding or metal–support interactions.^63,64^

For instance, RuSA@ Ti_3_C_2_T_*x*_ was fabricated by anchoring Ru single atoms onto Ti_3_C_2_T_*x*_*via* covalent bonding with surface oxygen (–O) groups, as shown in Fig. 6b, forming Ru–O–Ti bridges, with the Ru atoms uniformly distributed with a content of 0.44 wt%.^79^ This structure exhibits significantly enhanced HER performance (Fig. 6c–e), with a Tafel slope of 90 mV dec^−1^, compared to 44 mV dec^−1^ for the Pt/C reference catalyst. Supported by the Ti_3_C_2_T_*x*_ platform, Ru SAs serve as highly active centers for water splitting, accelerating the Volmer step, with the hydrogen binding energy reduced to 0.07 eV, close to the optimal value for H_2_ evolution. Furthermore, the turnover frequencies (TOFs) of RuSA@ Ti_3_C_2_T_*x*_, shown in Fig. 6e, indicate equivalently good performance with other SA-based cathodes. The TOF of RuSA@Ti_3_C_2_T_*x*_ increases exponentially with increasing cathodic potentials, indicating superior catalytic capability at high current densities. This highlights the potential of the Ti_3_C_2_T_*x*_ support in efficiently utilizing quantum effects to improve catalytic performance.^79^

Furthermore, the integration of surface engineering with single-atom composites has emerged as a promising strategy for enhancing HER catalysis. Gong *et al.*^71,80^ developed Pt single atoms on Ti_3_C_2_T_*x*_ (0.84 wt%) *via* a thermal shock method that simultaneously generated oxygen vacancies, which are favorable anchoring sites that stabilized Pt atoms and enabled exceptional performance, including a low overpotential of 38 mV at 10 mA cm^−2^, a Tafel slope of 45 mV dec^−1^, and a near-optimal Gibbs free energy of 0.02 eV for hydrogen adsorption.

Bifunctional systems of SAs have been explored to enhance electrocatalysts recently regarding Ti_3_C_2_T_*x*_ -MXene. Herein, Guo *et al.*^74^ proposed a composite of Co/Co_3_O_4_/Ti_3_C_2_T_*x*_; in this design, Ti_3_C_2_T_*x*_ serves as a stable scaffold offering numerous attachment sites for Co/Co_3_O_4_ and forms a highly conductive network that facilitates rapid electron transfer during electrocatalysis. Co_3_O_4_ has an important role in enhancing hydrophilicity, thereby accelerating bubble release. Meanwhile, the Co/Co_3_O_4_ phases promote H_2_O adsorption and dissociation, boosting H_2_ production. As a result, the composite exhibits excellent HER activity and durability in 1 M KOH, achieving an overpotential of 87 mV and a Tafel slope of 61.9 mV dec^−1^ at 10 mA cm^−2^.

**Fig. 7:**
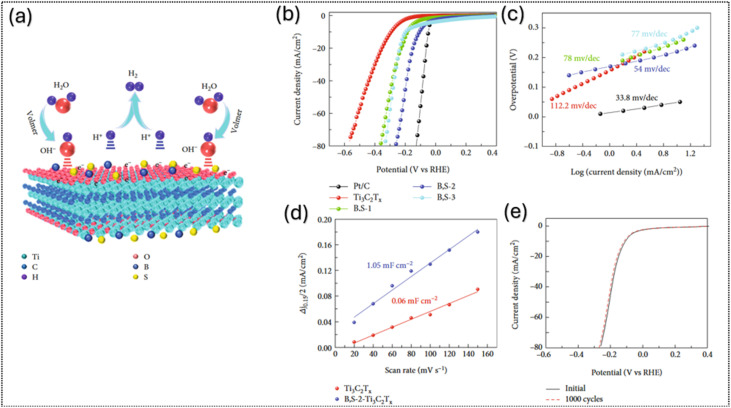
(a) Graphical illustration of the HER catalysis mechanism for B, S – Ti_3_C_2_T_*x*_ in an acidic medium; electrochemical performances of the as-prepared catalysts in 0.5 M H_2_SO_4_ with (b) LSV curves, (c)Tafel plot, (d) double-layer capacitance (*C*_dl_) at 0.15 V *vs.* RHE as a function of the scan rate and (e) polarization curves of B, S – Ti_3_C_2_T_*x*_ catalysts before and after 1000 CV cycles (at a scan rate of 50 mV s^−1^). Reproduced under the terms of the CC-BY license.^76^ Copyright 2023, Wiley.

Heteroatom doping with non-metals such as nitrogen, boron, or sulfur can improve MXene catalysts by changing their electronic structure, creating lattice defects, and adding new active sites. In 2023, Kim and co-workers^76^ used a one-step hydrothermal process to prepare boron and sulfur co-doped Ti_3_C_2_T_*x*_ (B,S–Ti_3_C_2_T_*x*_) from Ti_3_AlC_2_, as shown in Fig. 7. B and S co-doping in Ti_3_C_2_T_*x*_ redistributes charge and creates active sites: B–O–Ti and B–O–B sites adjust the electron density of Ti–C to favor H* formation in the Volmer step, while S sites promote proton/electron transfer and stabilize intermediates, accelerating H_2_ generation, as shown in Fig. 7a. Characterization revealed that Raman spectra confirmed more defects, and XPS showed electron transfer from Ti to B while DFT calculations indicated lower hydrogen adsorption free energy than that of undoped MXene about 0.087 eV. The catalyst reached an overpotential of 110 mV at 10 mA cm^−2^ with a Tafel slope of ∼54 mV dec^−1^, with the improvements attributed to the combined effects of boron, sulfur, and Ti_3_C_2_T_*x*_ in accelerating HER steps, as shown in Fig. 7b and c. Electrochemical tests showed that the electrochemical double-layer capacitance (*C*_dl_) which is often used to estimate the electrochemically active surface area of the catalyst B,S – Ti_3_C_2_T_*x*_ has *C*_dl_ more than 10 times higher than that of pristine Ti_3_C_2_T_*x*_, indicating that boron and sulfur doping significantly increase the number of accessible active sites (Fig. 7c); additionally, the sample does not show significant differences before and after cycling, exhibiting excellent HER activity with long-term stability over 1000 cycles (Fig. 7d). This study demonstrates a promising strategy for the metal-free co-doping of MXene nanosheets.

### 4.2 Oxygen evolution reaction

**Fig. 8:**
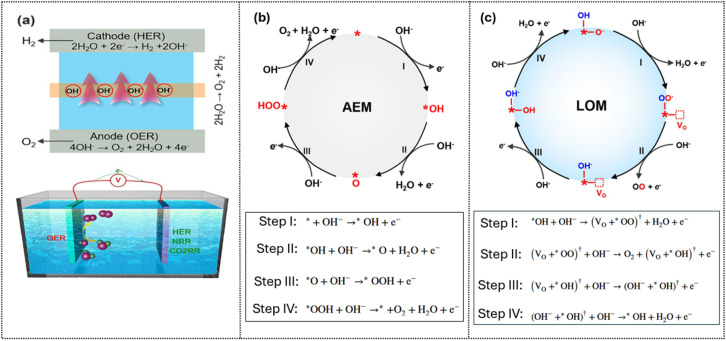
Illustration of the OER and mechanisms in electrocatalysis: (a) paired with the HER in electrocatalysis; (b) OER catalytic cycle based on the adsorbate evolution mechanism (AEM), and (c) lattice oxygen–mediated mechanism (LOM). Reproduced under the terms of the CC-BY-4.0 license.^81^ Copyright 2022, Springer.

The OER is the anodic half-reaction coupled with the HER in overall water splitting and in electrochemical processes such as the nitrogen reduction reaction (NRR) and CO_2_ reduction reaction (CO_2_RR) (Fig. 8a). The OER typically follows the 4e^−^ pathway involving the adsorbate evolution mechanism (AEM) or lattice oxygen mechanism (LOM). As the reaction happens to be based on the linear scaling relationship between Δ*G*_OH_, Δ*G*_O_, and Δ*G*_OOH_, and the binding energies of *OH and *OOH are strongly correlated, this imposes a theoretical overpotential limit of 0.37 V when compared with the ideal energy gap of 1.23 V.^60,81^

In the AEM, water or hydroxide is oxidized at active sites (*) through sequential formation of intermediates before releasing O_2_ as described in Fig. 8b with *OOH formation as the rate-determining step. As in Fig. 8c, the lattice oxygen mechanism (LOM) eliminates the concerted proton–electron transfer steps as occurred in the conventional AEM, which allows overcoming the AEM scaling limit. Based on direct lattice oxygen participation, such as *O radical formation, lattice–adsorbate O–O coupling, and oxygen vacancy this mechanism does not involve *OOH, so it is positioned as a route to break AEM scaling constraints. In the LOM pathway, the lattice oxygen needs to be active enough to leave or segregate from the lattice and react with surface oxygen species by generating oxygen *via* direct O–O bond coupling in highly covalent oxides, where electronic states near the Fermi level have substantial O 2p character, which can be achieved through the defect-engineering route and enhanced metal–oxygen covalency. Although the LOM may lower overpotential and boost intrinsic activity, it can also lead to instability through oxygen vacancy accumulation, metal dissolution, and lattice collapse.^81,82^

Another approach that is introduced to surpass the thermodynamic limitation of AEM overpotential is the oxide pathway mechanism (OPM). In this route, O–O bond formation occurs *via* direct coupling of two adjacent surface oxo and oxygen-radical species as intermediates, without generating the *OOH intermediate. By removing the *OH–*OOH scaling constraint, this pathway does not require lattice-oxygen participation or an oxygen-vacancy cycle as in the LOM pathway; instead, two surface *O species couple directly to release oxygen. This mechanism is important in water electrolysis because it is targeting the main efficiency bottleneck of energy loss in the OER. However, this pathway requires strict engineering to ensure stable, symmetric adjacent active sites with a narrowly defined inter-site distance to enable a low barrier for oxygen–oxygen coupling, as well as verification by *operando* characterization for mechanistic validation.

Ti_3_C_2_T_*x*_ MXene is an attractive OER platform due to its metallic conductivity, large surface area, and tunable terminations. However, it requires modification to improve binding with OER intermediates, increase active sites, and enhance stability against oxidation to TiO_2_ at anodic potentials.^60^ Strategies to improve Ti_3_C_2_T_*x*_ for the OER include electronic structure tuning based on heteroatom doping or defect engineering to optimize adsorption energies toward the volcano plot apex.^60,83^ Another approach is to integrate lattice-oxygen-active oxides with Ti_3_C_2_T_*x*_ to form Ti_3_C_2_T_*x*_/TiO_2_ heterojunctions, enhancing both activity and conductivity, or to focus on surface oxygen functionalities to increase active oxygen sources and promote proton–electron transfer steps.^60,83^

**Fig. 9:**
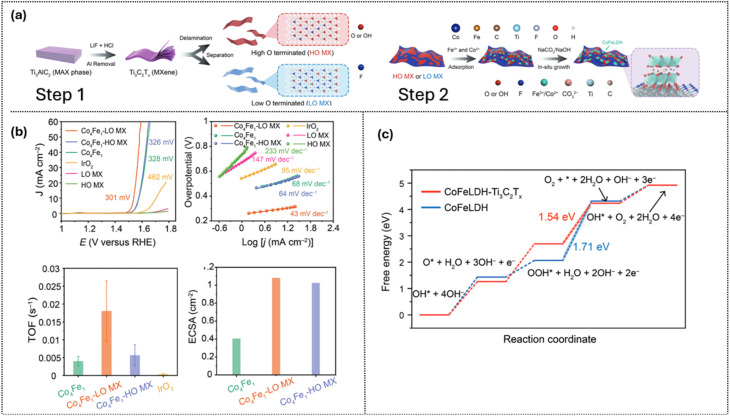
(a) Schematic of surface termination engineering of CoFe-LDH/Ti_3_C_2_T_*x*_, (b) paired with HER electrocatalysts, (c) electrochemical results from (b), and (d) DFT energy diagram showing reduced energy barriers for OER steps. Reproduced under the terms of the CC-BY license.^84^ Copyright 2025, Wiley-VCH.

Heterostructure engineering is a state-of-the-art strategy to optimize Ti_3_C_2_T_*x*_ -based OER catalysts by enhancing conductivity, stability, and activity at the interface. Sheng *et al.*^84^ developed a scalable, *in situ* guided growth of CoFeLDH while controlling the oxygen ratio on the surface of Ti_3_C_2_T_*x*_ to control the LDH crystal domain size. Fig. 9a displays the procedure to synthesize the composite of CoFeLDH/Ti_3_C_2_T_*x*_ with step 1 tailoring high oxygen (HO MX) or low oxygen (LO MX) and step 2 growing CoFe-LDH with Ti_3_C_2_T_*x*_. O-rich terminations on Ti_3_C_2_T_*x*_ guide LDH nucleation into smaller domains, increasing edge and defect sites for the AEM pathway. Oxygen terminations (–O and –OH) on MXene act as anchoring sites; HO MX has a higher density of O terminations, leading to more nucleation points and smaller LDH nanoplates, whereas LO MX has fewer O terminations, which means fewer nucleation sites but stronger interfacial coupling and electronic interaction per nucleation point. As shown in Fig. 9b, CoFe-LDH/LO MX delivers the smallest Tafel slope (43 mV dec^−1^), the highest turnover frequency (TOF), and the largest electrochemical surface area (ECSA), indicating more efficient active sites and greater accessibility. CoFe-LDH/Ti_3_C_2_T_*x*_ reduces the rate-determining step of the *OOH formation barrier from 1.71 eV (CoFe-LDH) to 1.54 eV *via* interfacial charge transfer, optimizing intermediate adsorption energies (Fig. 9c). DFT calculations show electron transfer from MXene to γ-CoFeLDH, lowering the rate-limiting barrier through interfacial charge redistribution, domain-size control, and high conductivity, resulting in stable performance in alkaline media and enhanced OER durability in water electrolyzers. The study demonstrates that termination control is a powerful strategy for tailoring nucleation, optimizing interfaces, and enhancing the overall durability of MXene-based OER catalysts.

**Fig. 10:**
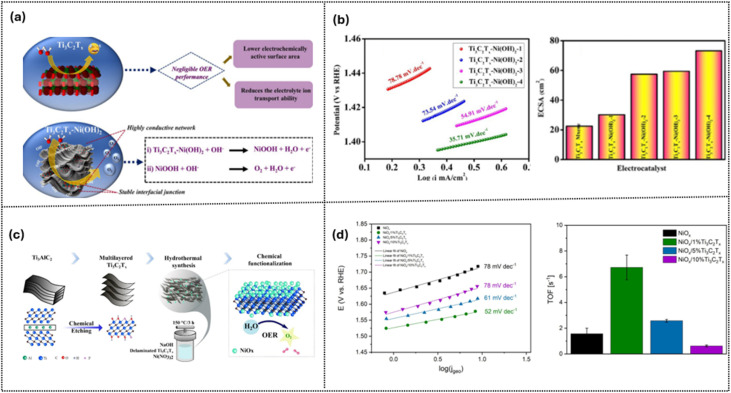
Ni–Ti_3_C_2_T_*x*_ electrocatalysts for the OER: (a) proposed reaction mechanism, (b) Tafel slopes and ECSA at varying Ni loadings. Reproduced under the terms of the CC-BY license.^86^ Copyright 2025, Wiley-VCH. (c) Schematic synthesis route, and (d) Tafel slopes and TOF at varying Ti_3_C_2_T_*x*_ contents. Reproduced under the terms of the CC-BY license.^85^ Copyright 2025, Wiley-VCH.

Transition metal oxides have emerged as promising OER catalysts due to their feasibility in large-scale production, with Ni-based materials having been extensively studied for their enhanced OER activity.^85^ This is because coupling Ni(OH)_2_ or NiO_*x*_ with Ti_3_C_2_T_*x*_ forms a conductive and stable interface that enhances electron transport and active site accessibility.^85,86^ Browne's team^85^ in Germany and Oh's team^86^ in Korea independently studied how the Ni/Ti_3_C_2_T_*x*_ ratio affects OER performance (Fig. 10). Bare Ti_3_C_2_T_*x*_ shows negligible activity due to a low ECSA and poor intermediate binding, but coupling it with Ni(OH)_2_ or NiO_*x*_ forms a conductive, stable interface that improves electron transport and active site accessibility (Fig. 10a). Increasing the Ni(OH)_2_ loading on Ti_3_C_2_T_*x*_ enhances the ECSA, lowers the Tafel slope (from 78 to 35 mV dec^−1^), and accelerates the OER kinetics (Fig. 10b). Ti_3_C_2_T_*x*_ also promotes the α → β phase transformation in Ni(OH)_2_, with β-NiOOH as the active phase, and facilitates faster deprotonation in the AEM pathway. However, the lowest ratio of MXene loading (1 wt%) exhibits the highest TOF by balancing electron transport and Ni site exposure, while excessive loading reduces activity due to site blockage and TiO_2_ formation (Fig. 10d). These findings underscore the importance of balancing the Ni-MXene ratio to maximize catalytic performance and ensure long-term stability for efficient OER catalysis.

Bifunctional catalysts capable of promoting both the HER and OER have been researched as a practical route toward overall water splitting for hydrogen production. This approach enables simplified electrolyzer architecture and reduces materials costs based on matched electrode kinetics.

In practice, bifunctional HER-OER catalysts for overall water splitting have attracted great attention for combining the merits of both reactions. A symmetric design operating in the same electrolyte simplifies the system, reduces energy consumption and cost, and enables scalable applications. An ideal bifunctional system requires high activity and stability in the same pH environment for both the HER and OER, along with balanced binding energies for hydrogen- and oxygen-containing intermediates. The main challenge is to minimize overpotentials at both electrodes while meeting the optimal surface chemistry requirements of both reactions and maintaining stability at highly reducing and oxidizing potentials in the same electrolyte.^87,88^ Regarding this challenge, Ti_3_C_2_T_*x*_ is integrated as a platform to address multiple bottlenecks, including improving carrier pathways to accelerate the HER and OER kinetics, tuning surface terminations to adjust adsorption energy, and hybridizing with active phases to achieve optimal binding energies. Its 2D structure can anchor nanoparticles, single atoms, or molecular catalysts, preventing aggregation and enhancing stability under redox cycling.^70,89,90^

**Fig. 11:**
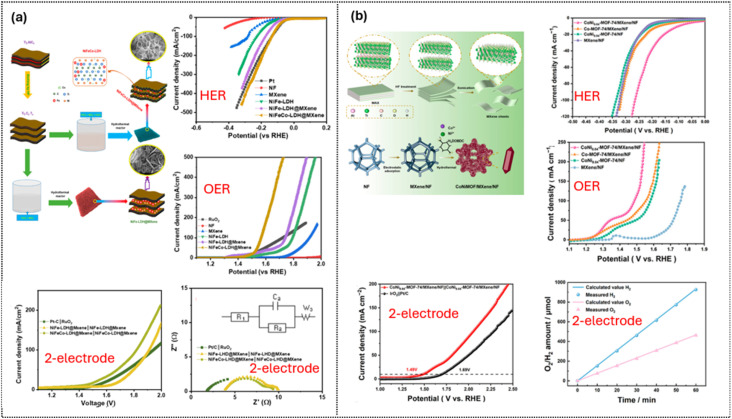
Ti_3_C_2_T_*x*_-based composites for efficient overall water splitting: (a) synthesis illustration and the electrocatalytic performances of Ti_3_C_2_T_*x*_-supported NiFeCo-LDH. Reproduced under the terms of the CC BY 4.0 license.^91^ Copyright 2022, MDPI. (b) Synthesis illustration and the electrocatalytic performances of the Ni-doped Co-MOF-74/Ti_3_C_2_T_*x*_ MXene composite and its electrocatalytic performance. Reproduced under CC BY 4.0.^92^ Copyright 2024, MDPI.

An example of prominent studies in this direction is the three-dimensional honeycomb-like structure of NiFeCo-LDH on Ti_3_C_2_T_*x*_ reported by Hussain *et al.*^91^Fig. 11a illustrates the synthesis and electrocatalytic performance of NiFeCo–LDH@Ti_3_C_2_T_*x*_ MXene on Ni foam. The Ti_3_C_2_T_*x*_ scaffold prevents LDH aggregation, exposes more active sites, and maintains open interlayer channels for ion diffusion, forming a honeycomb-like porous structure. Direct growth of NiFeCo–LDH on MXene yields a conductive, hydrophilic electrode with strong interfacial coupling that enhances charge transfer. In the HER, the composite achieves a low overpotential of 34 mV, outperforming all controls. In the OER, it reaches 130 mV with a Tafel slope of 52 mV dec^−1^ and a steeper LSV rise than MXene or NiFe-LDH alone. In overall water splitting, the symmetric NiFeCo-LDH@MXene‖NiFeCo-LDH@MXene cell delivers 10 mA cm^−2^ at 1.41 V, lower than that in Pt/C‖RuO_2_ (1.75 V). EIS shows that a smaller semicircle corresponds to faster electron transfer and lower charge-transfer resistance, with NiFeCo-LDH@MXene exhibiting the lowest *R*_ct_ of 4.6 Ω, confirming superior kinetics and outstanding stability during 24 h of testing. This underscores the synergistic effect of Ti_3_C_2_T_*x*_ in enhancing electron transport, optimizing intermediate adsorption, and stabilizing the structure under redox conditions.

Another innovation was reported by Yu *et al.*^92^ with the design of a composite electrode combining nickel foam (NF), Ti_3_C_2_T_*x*_ MXene, and a Ni/Co-based MOF. The resulting CoNi-MOF-74/MXene/NF acts as a bifunctional and durable water-splitting electrocatalyst. As shown in the LSV curves, the composite significantly outperforms Co-MOF-74/NF and MXene/NF controls: for the HER, it achieves an overpotential of 102 mV at 10 mA cm^−2^ with a Tafel slope of 123.5 mV dec^−1^, consistent with a Volmer-limited mechanism; for the OER, it reaches 100 mA cm^−2^ at 256 mV with a Tafel slope of 40.21 mV dec^−1^, reflecting fast oxygen evolution kinetics. In a two-electrode configuration using the same material at both electrodes, the system delivers 10 mA cm^−2^ at 1.49 V and operates stably for 40 h with a faradaic efficiency of 97% (H_2_ : O_2_ ≈ 2 : 1). Mechanistic studies indicate that Ni doping increases the Co^3+^/Co^2+^ ratio, generating more active sites, while strong MXene–MOF interfacial coupling lowers charge-transfer resistance and increases double-layer capacitance. After the OER, the catalyst surface partially converts to Co(Ni)OOH, which further optimizes adsorption of *H and O-intermediates and accelerates charge transfer. In both systems, Ti_3_C_2_T_*x*_ acts as a conductive, wettable scaffold that prevents restacking/aggregation, exposes more edge sites, eases Volmer water activation (HER) and OOH formation pathways (OER), and manages gas release, making the composites clearly outperform their individual precursors under practical alkaline conditions.

Ti_3_C_2_T_*x*_ MXene has emerged as an effective platform for seawater electrolysis by mitigating the competing chlorine evolution reaction (CER) and stabilizing catalysts in chloride-rich environments, which is not ideal for the HER/OER as it competes with the OER, and chloride ions corrode active sites by adsorbing on polarized electrodes. The high conductivity of Ti_3_C_2_T_*x*_ lowers the OER overpotential, making the OER kinetically favorable over the CER, while its tunable surface terminations preferentially stabilize oxygen intermediates rather than chloride ions. In addition, its 2D layered framework provides abundant ion channels and anchors transition-metal nanoparticles or single atoms, preventing aggregation and shielding active sites from chloride attack.^93,94^ For example, leveraging the intrinsic properties of NiFe-LDH to repel chloride ions during seawater oxidation,^95^ and when integrated with Ti_3_C_2_T_*x*_, the strong Ti–O–Fe bonding, electron withdrawal, and surface charge synergistically suppress corrosion and Cl^−^ attack while enhancing the lattice-oxygen-driven OER, enabling highly efficient and durable seawater electrolysis.^96^

Table 1 overviews the latest innovations for the HER, OER, and water splitting, with ideas summarized by focusing on non-noble metal alternatives, maximizing the efficiency of quantum catalysts from SA structures, structure engineering through doping, heterostructures, surface modifications, and combining these strategies with excellent performance data. The overall goal is to achieve the lowest overpotential and Tafel slope, which indicate highly active catalytic sites, while also retaining structural stability for long-term use and keeping the approach open for scalable applications.

**Table 1:** Summary of Ti_3_C_2_T_*x*_-based catalysts for HER, OER, and overall water-splitting applications

Ti_3_C_2_T_*x*_ composites	Electrolyte	Overpotential [mV]	Tafel slope [mV dec^−1^]	Application	Ref.
Pristine Ti_3_C_2_T_*x*_	0.5 M H_2_SO_4_	300	86.6	HER	76
Pristine Ti_3_C_2_T_*x*_	1 M KOH	541	324.46	HER	74
PtSA-Ti_3_C_2_T_*x*_	0.5 M H_2_SO_4_	38	45	HER	80
RuSA-Ti_3_C_2_T_*x*_	1 M KOH	40.3	90	HER	79
Co/Co_3_O_4_–Ti_3_C_2_T_*x*_	1 M KOH	87	61.9	HER	74
B, S–Ti_3_C_2_T_*x*_	0.5 M H_2_SO_4_	110	54	HER	76
CoP–Ti_3_C_2_T_*x*_	0.5 M H_2_SO_4_	135	48	HER	73
Fe@Ti_3_C_2_T_*x*_	1 M KOH	81	33.03	HER	75
Pristine Ti_3_C_2_T_*x*_	1 M KOH	520	68	OER	91
Ni(OH)_2_–Ti_3_C_2_T_*x*_	1 M KOH	235.54	35.71	OER	86
CoP@C – Ti_3_C_2_T_*x*_	1 M KOH	235	54	OER	97
Ni–Ti_3_C_2_T_*x*_	1 M NaOH	354	54	OER	85
CoFeLDH-Ti_3_C_2_T_*x*_	1 M KOH	301	43	OER	84
CuS@Ti_3_C_2_T_*x*_	1 M KOH	89.7	54	HER	98
		171	39.3	OER	
NiFeCo–LDH@ Ti_3_C_2_T_*x*_	1 M KOH	34	62	HER	91
		130	52	OER	
CoSe_2_–Ti_3_C_2_T_*x*_	1 M KOH	230	65	HER	70
		270	71	OER	
CoNi-MOF-74/Ti_3_C_2_T_*x*_/NF	1 M KOH	102	123.5	HER	92
		256	40.2	OER	

### 4.3 Gas conversion reactions

Recently, there have been calls for reducing greenhouse gas emissions to tackle the global climate change. And the innovation of gas conversion to value products such as chemicals or fuels is emerging as a hot topic in the catalyst field. Electrocatalytic gas reduction enables the direct conversion of molecules such as CO_2_ (CO_2_RR), NH_3_ (NRR), and O_2_ (ORR) to value-adding products as chemicals or fuels. However, conventional catalysts often suffer from low selectivity, slow kinetics, poor stability, harsh operating conditions and reliance on expensive noble metals.^99–103^ To address these limitations, Ti_3_C_2_T_*x*_ has emerged as a promising candidate, enabling adsorbate pathways where rates and selectivity are controlled by surface terminations, anchored active sites, and coverage effects that link co-adsorbates to otherwise unfavorable intermediates.^104^

**Fig. 12:**
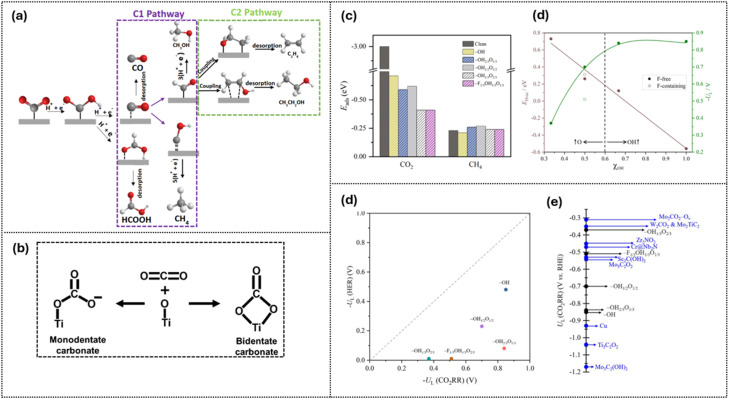
The illustration of the theoretical model and calculations regarding Ti_3_C_2_T_*x*_ for the CO_2_RR: (a) C_1_*vs.* C_2_ pathways for CO_2_ electro-reduction. Reproduced under the terms of the CC-BY license.^105^ Copyright 2023, MDPI. (b) Proposed CO_2_ adsorption geometry on the MXene surface. Reproduced under the terms of the CC-BY license^102^ Copyright 2023, RSC. (c) CO_2_ and CH_4_ adsorption energies corresponding to various terminations of Ti_3_C_2_T_*x*_, (c) H-vacancy energies and limiting potential in terms of oxygen groups of F-controlling Ti_3_C_2_T_*x*_, (d) comparision of limiting potential (UL) of the HER and CO_2_RR, (e) comparison of UL of methane from Ti_3_C_2_T_*x*_ with other candidates. Reproduced under the terms of the CC-BY-NC license.^106^ Copyright 2024, RSC.

A major focus in gas reduction is the CO_2_ reduction reaction (CO_2_RR), which converts CO_2_ into value-added C_1_ and C_2_^+^ products such as methanol, ethanol, ethylene, and acetone. This process faces two key challenges: the insufficient adsorption and activation of intermediates on many electrodes, and strong competition from the hydrogen evolution reaction. On Ti_3_C_2_T_*x*_ MXene, the CO_2_RR proceeds through proton-coupled electron transfer (PCET) steps involving complex intermediates that depend on the electrocatalyst and reaction conditions. Intermediates formed during the CO_2_RR through proton–electron transfer steps yield C_1_ products (*e.g.*, CO, CH_4_, CH_3_OH, and HCOOH), while further C–C coupling of CO species leads to C_2_ products (*e.g.*, C_2_H_4_, CH_3_CH_2_OH, and CH_3_COOH).^105^ In this context, a DFT modeling study by Lozano-Reis and Exner^106^ emphasized the critical role of less-stable intermediates and co-adsorbates in determining CO_2_RR selectivity on Ti_3_C_2_T_*x*_, particularly through tunable surface terminations. The active Ti sites facilitate CO_2_ adsorption, initially forming carbonate-like intermediates where monodentate binding is less stable than bidentate, thereby directing distinct reduction pathways as shown in Fig. 12.

Importantly, adsorption strength must strike a balance: CO_2_ should bind strongly enough for activation but not so strongly as to cause surface poisoning, while desired products such as CH_4_ require sufficiently weak binding to ensure efficient release and avoid catalyst deactivation. The adsorption-energy trends in Fig. 12c show that CH_4_ binding is already suitable, but CO_2_ adsorption on clean Ti_3_C_2_T_*x*_ is too strong. Introducing mixed –O/–OH/–F terminations moderates CO_2_ binding into a more favorable activation regime.^106^ Termination-controlled thermodynamics further influence the required driving force, as shown in Fig. 12d, with the computed limiting potential (UL) varying with the –OH fraction (*χ*_OH_). The results indicate that a balanced presence of –OH and –O groups, with a small proportion of F, optimizes both the limiting potential and proton transfer rate.^106^ Consistently, Exner *et al.*^104^ reported that the highest selectivity is observed for CH_3_OH and CH_4_ at low to medium F coverage, with CH_4_ dominating at higher F levels. Since the HER is a key competing pathway, Fig. 12d also displays the comparison of UL(CO_2_RR) with UL(HER), identifying termination windows where the CO_2_RR is thermodynamically favored. This demonstrates how mixed terminations provide a practical lever for selectivity control. Relative to other catalyst families, Ti_3_C_2_T_*x*_ with optimized O/OH terminations achieves competitive CO_2_RR limiting potentials, comparable in magnitude to established Cu- and Mo-based systems, while maintaining the unique advantage of tunable surface chemistry on a highly conductive 2D scaffold (Fig. 12e). This demonstrates that Ti_3_C_2_T_*x*_ is not only tunable but also potentially performs comparably to state-of-the-art CO_2_RR catalysts.

Krishnan *et al.*^102^ demonstrated that Ti_3_C_2_T_*x*_ nanosheets enhance CO_2_RR performance by offering a highly conductive 2D scaffold with abundant surface terminations that promote CO_2_ adsorption and activation. The nanosheets displayed strong CO_2_ affinity (0.16 mmol g^−1^ uptake at 298 K) and high electrochemical activity, evidenced by a large double-layer capacitance (*C*_dl_ = 3.19 mF cm^−2^), which correlates with more active sites. During electrolysis, the catalyst achieved a peak faradaic efficiency of 53.8% for liquid products and an overall efficiency of 96%, producing both C_1_ (CO and CH_3_OH) and C_2_^+^ products (C_2_H_5_OH and CH_3_COCH_3_) at −1.1 V *vs.* Ag/AgCl in 0.5 M KHCO_3_ before decreasing due to HER dominance. Stability testing over 72 h at 1.1 V confirmed negligible decay in current density and minimal structural degradation. Although Ti_3_C_2_T_*x*_ MXene exhibits excellent CO_2_ adsorption and stability, its limited selectivity, competition with the hydrogen evolution reaction, and complex surface termination structure underscore the need for refined design strategies to enhance its efficiency for CO_2_ electroreduction.

Recent advances position Ti_3_C_2_T_*x*_-based MXenes as versatile platforms for the CO_2_RR, with composites like oxides, COF hybrids, and single-atom decorations enhancing activity, selectivity, and rates.^107^ Formate, a valuable CO_2_RR product due to its energy content, can be efficiently produced using Ti_3_C_2_T_*x*_-based catalysts. Notably, SnO_2_ quantum dot decoration on Ti_3_C_2_T_*x*_ enables highly selective formate generation, achieving approximately 94% faradaic efficiency and a partial current density of 57.8 mA cm^−2^. This performance is attributed to reduced interfacial charge-transfer resistance and dynamic modulation of the Sn oxidation state under applied potential, as revealed by *in situ* synchrotron-based X-ray absorption spectroscopy (XAS).^108^

The development of a 3D Cu–Pd/Ti_3_C_2_T_*x*_ aerogel highlights the role of MXene in enhancing CO_2_ adsorption and expanding the electrochemically active surface area within a binder-free, porous architecture that integrates Cu–Pd bimetallic sites. Ti_3_C_2_T_*x*_ supports tuning of active sites and shifts selectivity from CO to formate by providing a lower-energy pathway for HCOO^−^ formation. Experimental results further show improved charge transfer, facilitated kinetics of the rate-determining step, and mitigation of the limitations of Cu–Pd alone. Consequently, the catalyst achieves an industrially relevant current density of 150 mA cm^−2^ with >90% FE for formate in a membrane electrode assembly, delivering ∼47% full-cell energy efficiency and stable operation for 5 hours.^109^

In a study from Zhao *et al.*,^110^ single-atom Cu anchored on Ti_3_C_2_Cl_*x*_ MXene acts as an effective support, where Cu–O bonding and Cl termination create a distinct pathway toward alcohols. XAS and DFT confirmed that the unsaturated electronic structure of Cu centers lowers the energy barrier for the HCOOH* to CHO* conversion, enabling 59.1% FE for methanol in an H-cell with commendable electrocatalytic stability.

**Fig. 13:**
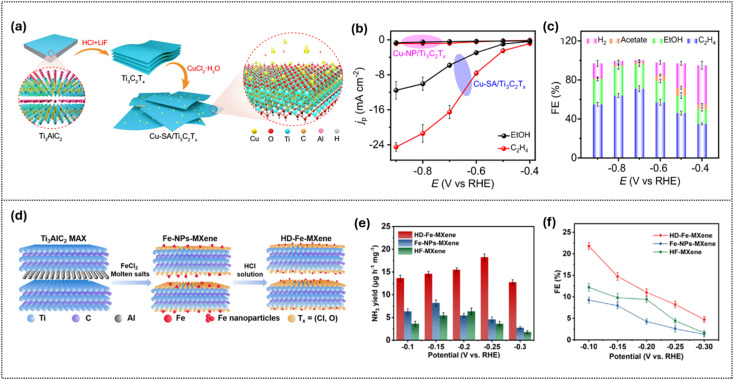
Synthesis and electrocatalytic performance of single-atom MXene catalysts for CORR and NRR applications. (a) Schematic preparation of Cu single-atom sites anchored on Ti_3_C_2_T_*x*_ and (b) corresponding partial current densities for C_2_H_4_ and EtOH production. (c) Faradaic efficiencies of H_2_ and CO_2_ reduction products on Cu-SA/Ti_3_C_2_T_*x*_ at different potentials. Reproduced under the terms of the CC BY 4.0 license.^100^ Copyright 2021, Nature Communications. (d) Controlled etching strategy to immobilize Fe atoms in MXene, forming HD-Fe-MXene, and (e) NH_3_ yields and (f) faradaic efficiencies at different potentials. Reproduced under the terms of the CC-BY license.^111^ Copyright 2022, Wiley.

In the trend of gas-phase electrocatalysis, studying the CO reduction reaction (CORR) is an effective strategy instead of the CO_2_RR, when targeting the crucial C–C coupling stage of the CO_2_RR by bypassing the rate-limiting CO_2_ → CO step and avoiding competing H_2_ or HCOOH pathways. Bao *et al.*^100^ demonstrated that anchoring Cu SAs onto Ti_3_C_2_T_*x*_ as isolated Cu–O_3_ single sites enables highly efficient C_2_^+^ production, including ∼71% faradaic efficiency (FE) for ethylene and ∼25% for ethanol, retaining stability for 68 h. As shown in Fig. 13a, the catalyst is synthesized by exfoliating Ti_3_AlC_2_ into Ti_3_C_2_T_*x*_ and anchoring single Cu atoms, forming well-defined atomic Cu–O_3_ sites. This design yields outstanding results. Correspondingly, Fig. 13b shows that partial current densities for C_2_H_4_ (−16.5 mA cm^−2^) and EtOH (−5.8 mA cm^−2^) on Cu–SA/Ti_3_C_2_T_*x*_ far exceed those of Cu nanoparticles, highlighting the role of atomically dispersed sites in accelerating C–C coupling. Finally, Fig. 13c confirms the product distribution across applied potentials, with C_2_H_4_ dominating, EtOH as the major liquid product, and the HER strongly suppressed. DFT calculations attribute this performance to the stabilization of the *CO–CHO intermediate at Cu–O_3_ sites, lowering the energy barrier for C–C coupling and explaining the high activity and durability relative to nanoparticle counterparts.

For nitrogen chemistry, Ti_3_C_2_T_*x*_ enables efficient N_2_ activation when active sites and terminations are co-optimized. Embedding highly dispersed Fe in fluorine-free Ti_3_C_2_T_*x*_*via* MAX-replacement plus HCl etching yields FE(NH_3_) = 21.8% at −0.10 V and an NH_3_ yield of 18.25 µg h^−1^ mg^−1^ at −0.25 V in 0.1 M Na_2_SO_4_ with 24 h/multicycle stability, evidencing that F-removal exposes/immobilizes Fe while curbing the HER. Extending to nitrate reduction, a cross-MXene study finds that Ti_3_-based members (including Ti_3_C_2_T_*x*_) lead their class in FE, yield, and stability, with DFT tracing performance to central-metal identity and T_*x*_-controlled adsorption energetics. Together these results define the state of the art: termination/coverage engineering plus site design (dispersed metals or molecular centers) on conductive Ti_3_C_2_T_*x*_ provides coherent levers to tune activity, selectivity, and durability across CO_2_RR and N-reduction pathways.^111^

The oxygen reduction reaction (ORR) is a gas-involving electrochemical reaction and is considered the thermodynamic reverse of the OER. In the electrocatalysis field, the ORR governs cathodic performance in fuel cells and metal–air batteries. The ORR involves O–O bond breaking, dominated by adsorption energetics at isolated surface sites. The adsorption-controlled mechanism involves surface-bound oxygen intermediates (*O_2_, *OOH, *O, and *OH) with O_2_ being sequentially reduced and protonated until the O–O bond is completely cleaved into water or hydroxyl molecules. As the reactions are governed by the adsorption energy of these intermediates, especially *OOH, this results in constraints on strong scaling relationships. However, lattice oxygen participation or oxygen-vacancy dynamics, which can be employed in the OER, does not play a role in the ORR, making breaking of scaling relations much harder in the ORR.^112^

MXenes with the synergetic effects based on metal-like conductivity, tunable surface terminations, and strong interaction with active species have been proposed as a potential candidate for ORR applications with the ability to act as conductive supports, electronic modulators, or anchoring substrates for catalytically active centers. Specifically, oxygen-containing terminations such as –O and –OH can enhance O_2_ and *OOH stabilization, shifting the free-energy profile closer to optimal values as predicted by adsorption volcano relationships. MXenes serve as effective supports for SACs and heteroatom-doped motifs for preventing metal aggregation and tuning the d-band center, which can improve the ORR performance. In other words, the electronic environment of the active site is optimized by the MXene substrate.^112^

The state of the art now points toward rational control of termination chemistry, atomic dispersion, and device-level architectures to bridge lab-scale activity with practical gas electroreduction, while open questions remain regarding long-term durability, synthesis scalability, and controlling selectivity for specific desirable products. More ideas on Ti_3_C_2_T_*x*_-based electrocatalysts for gas reduction are summarized in Table 2.

**Table 2:** Summary of Ti_3_C_2_T_*x*_-based catalysts for gas reduction reactions

Ti_3_C_2_T_*x*_ composite/system	Reaction	Best metric (FE or rate)	Main products/selectivity	Ref.
Ti_3_C_2_T_*x*_	CO_2_RR	FE: 96%	CO 42.2%, CH_3_OH 23.6%, EtOH 20.1%, acetone 10.1%	102
CoPc/o–N–MXene	CO_2_RR	FE: 39.5%	CH_3_OH	113
Cu single atoms on Ti_3_C_2_T_*x*_	CORR	FE: 71%	C_2_H_4_	100
Cu–Pd 3D Aerogels/Ti_3_C_2_T_*x*_	CO_2_RR	FE: 79%	Formate	109
HD-Fe-MXene	N_2_RR → NH_3_	FE: 21.8%	NH_3_	111
Ti_3_C_2_T_*x*_	NO_3_RR → NH_3_	FE: 5.78%	NH_3_	114
FeN_4_/Ti_3_C_2_T_*x*_	ORR (alkaline)	N/A	H_2_O (4e^−^)	112
(FePc/Ti_3_C_2_T_*x*_)			H_2_O_2_ < 1%	

## 5. Conclusions and future perspectives

Ti_3_C_2_T_*x*_ MXene has been emerging over the last 5 years, with most results still in theoretical studies. This MXene attracts attention as a promising platform for electrocatalysis thanks to its metallic conductivity, 2D layered structure, and tunable surface terminations. The surface terminations, such as –O, –OH, and –F functional groups, strongly affect catalytic behavior by shaping adsorption energies and the electronic environment of active sites. For instance, –F groups are usually unfavorable for the HER because they hinder proton adsorption, but in the CO_2_RR they help suppress hydrogen evolution and stabilize intermediates, indirectly improving selectivity. Beyond its intrinsic activity, Ti_3_C_2_T_*x*_ is also valued as a conductive scaffold with a large surface area that supports charge-carrier transport and adsorption capacity, which is especially useful in gas reduction as it allows more space for gas bubbles during the reaction. It is capable of hosting catalytic centers through strategies such as defect engineering, heteroatom doping, single-atom anchoring, and heterostructure assembly. Ti_3_C_2_T_*x*_ is versatile, with different methods to develop composites and anchor single atoms from both vacancies and terminations. Such approaches have enabled notable advances, from atomically dispersed noble metals to the exploration of new catalysts based on non-noble transition metals or synergistic dual-metal systems, which allow the development of bifunctional HER and OER activity.

As the research field of Ti_3_C_2_T_*x*_ electrocatalysts is still in an early developmental phase, the potential for practical application is evident but remains constrained in practical studies. Ti_3_C_2_T_*x*_ catalysts often require higher overpotentials than noble metals, suffer from oxidation and restacking under harsh conditions, and lack precise control over surface terminations due to current etching methods. Scalability and long-term durability at industrial current densities also remain open questions, with many designs still at the theoretical or proof-of-concept stage. A persistent challenge lies in controlling surface terminations, as conventional etching routes often produce heterogeneous functional groups, leading to variability in catalytic behavior. Ti_3_C_2_T_*x*_ itself possesses limited intrinsic activity; thus, advancing MXene-based electrocatalysts requires strategies to increase active sites, such as vacancy engineering or doping, while maintaining conductivity and structural stability under harsh conditions, as well as optimizing adsorption and desorption of key intermediates. The scalability of synthesis also remains restricted, and long-term durability at industrially relevant current densities is rarely demonstrated, raising questions about practical viability. Addressing these obstacles includes lowering overpotentials to match noble metals, achieving precise termination engineering, ensuring oxidation resistance and structural stability, and developing green, scalable production methods, which will be essential for consistent performance in extended operation.

Based on the current state of the art and the requirement for scalability and cost-effectiveness, three directions stand out: first, surface engineering, such as surface termination, doping, or creating vacancies, that can better control adsorption and reactivity; second, advancing single-atom and dual-atom catalysis, ideally using earth-abundant elements for scalability while taking advantage of the unique properties of noble and transition metals; third, employing *operando* tools such as XAS, Raman, and XPS to track active sites under working conditions to verify the mechanism during the reaction. Synergetic ideas such as heterostructures, surface engineering, and surface anchoring are highly recommended to address electrocatalysts for specific cases and expand their applications. Alongside these, long-term durability tests and greener, scalable synthesis methods will be key for practical deployment.

In short, Ti_3_C_2_T_*x*_ has proven itself as a versatile scaffold for electrocatalytic applications such as the HER, OER, and CO_2_RR, where atomic-level design strategies already deliver impressive results. The next breakthroughs will come from mastering stability, termination control, and scalability, moving MXene-based catalysts from the lab toward real applications in sustainable energy conversion.

## Conflicts of interest

The authors declare that they have no competing financial interests or personal relationships that could have influenced the work reported in this study.

## Data Availability

No new data were created or analyzed in this study. All data supporting the findings of this work are available within the published literature cited throughout the manuscript.
